# A Likely Case of Abemaciclib-Induced Hyperpigmentation in a Patient With Metastatic Breast Cancer

**DOI:** 10.7759/cureus.28948

**Published:** 2022-09-08

**Authors:** Michela Salusti-Simpson, Hannah Porter, Keith Morley

**Affiliations:** 1 Dermatology, University of Vermont Medical Center, Burlington, USA

**Keywords:** to illustrate this rarely described cutaneous adverse effect of the drug, drug-reaction, adverse cutaneous drug reaction, breast cancer research, abemaciclib, cutaneous hyperpigmentation, cutaneous adverse drug reaction

## Abstract

We report a case of a 64-year-old female with a past medical history of invasive right breast adenocarcinoma presented with diffuse hyperpigmentation of her skin after admission to the hospital for an infected breast implant. She had no recollection of a similar cutaneous reaction in her past. The patient had been on a chronic regimen of anastrozole and abemaciclib for her metastatic breast cancer. A punch biopsy revealed results were highly suspicious for a drug-induced hyperpigmentation reaction. After a thorough review of the patient's current and past medication lists, it was determined that her abemaciclib was the most likely culprit of her hyperpigmentation. This case is significant because of the rarity of this possible specific cutaneous reaction to abemaciclib. The literature that exists on cyclin-dependent kinase 4 and 6 inhibitors (CDK 4/6) is minimal. And so, the importance of shedding light on its possible cutaneous side effects is not only helpful for clinician diagnosis but also essential for patients to make informed decisions. To our knowledge, there is no other published literature on likely abemaciclib-induced hyperpigmentation.

## Introduction

The use of targeted anti-cancer therapy has become an increasingly important component of breast cancer treatment. With increased utilization of this treatment type, new side effects are also recognized, including diverse cutaneous toxicities. The cyclin-dependent kinase 4 and 6 (CDK 4/6) inhibitors have come to play an important role in targeted anti-cancer therapy treatment for breast cancer [1]. While bone marrow suppression is a known side effect, rare cutaneous side effects have also been reported. The abemaciclib product monograph includes cutaneous side effects such as rash, alopecia, pruritis, and nail ridging [2]. Herein, we report a case of a 64-year-old female with metastatic breast cancer and subsequent development of hyperpigmentation suspected to be induced by the CDK 4/6 inhibitor abemaciclib. This was a diagnosis of exclusion with a thorough review done of the patient's medications and other possible triggers for her hyperpigmentation. The data reported on cutaneous side effects associated with CDK 4/6 inhibitors is minimal, thus we are hopeful that this report will help to raise awareness of hyperpigmentation as a possible side effect of these agents. To our knowledge, this specific cutaneous manifestation has not yet been reported in association with abemaciclib in the literature.

## Case presentation

**Figure 1:**
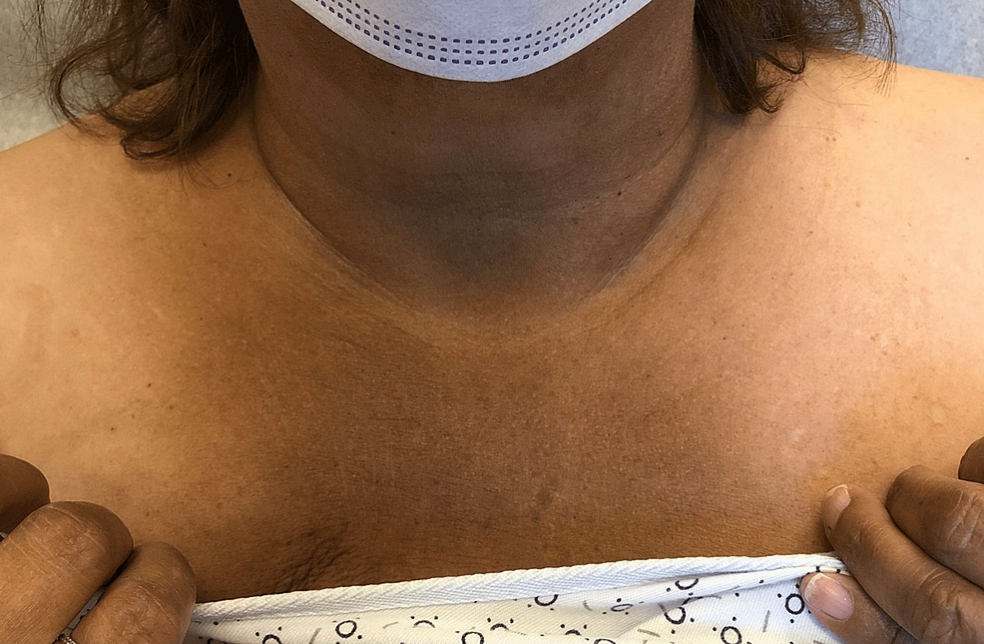
Hyperpigmented patch of the central chest and neck

**Figure 2:**
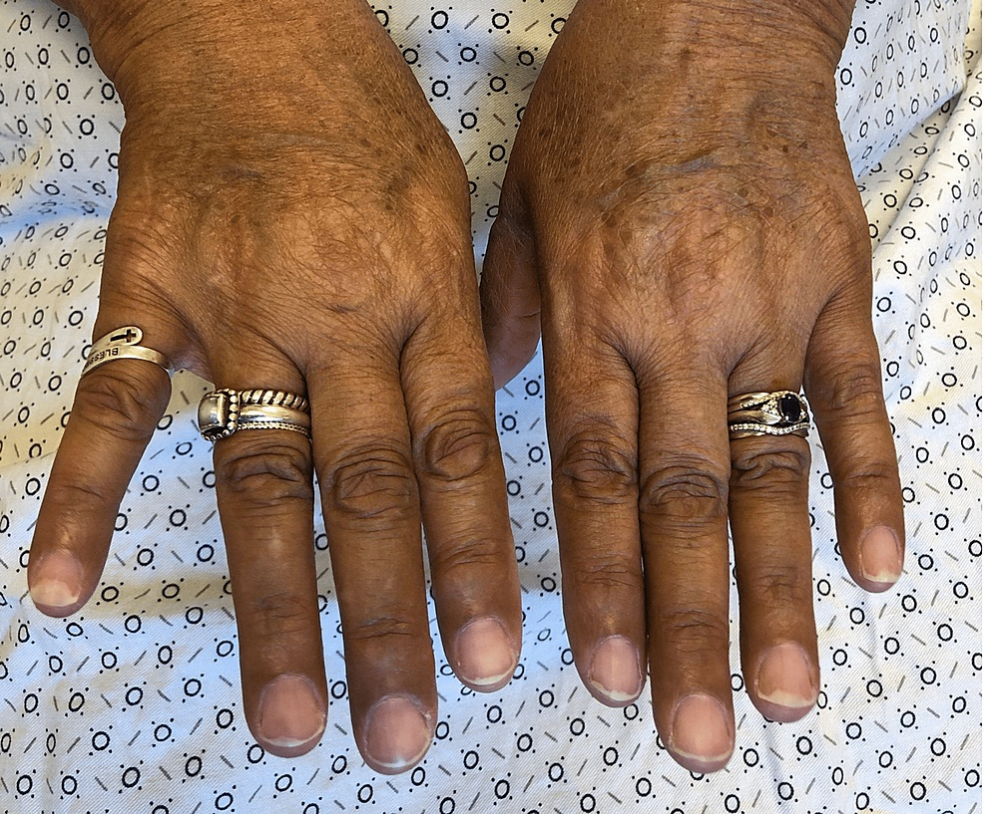
Diffuse hyperpigmentation of bilateral dorsum of hands and fingers

**Figure 3:**
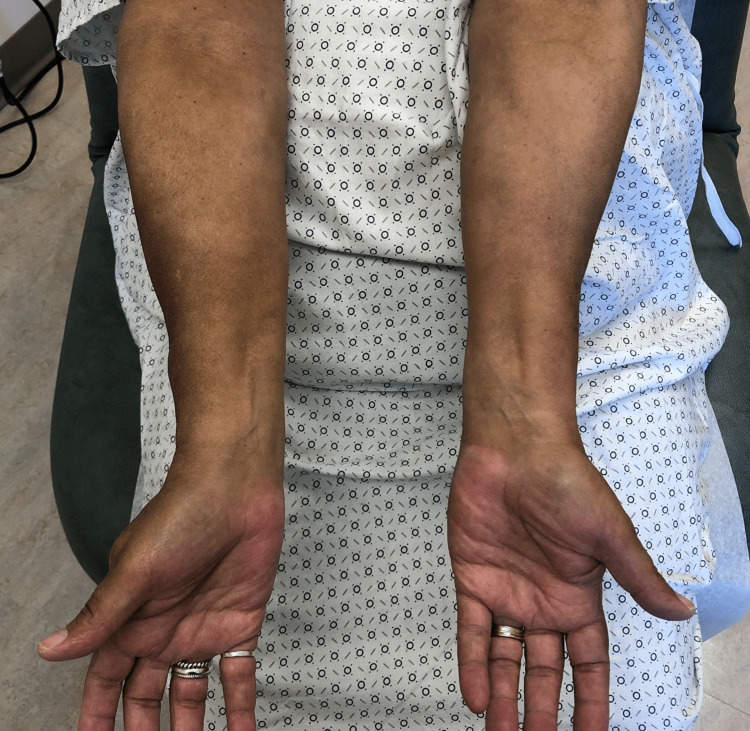
Hyperpigmented patches of bilateral ventral surfaces of forearms

**Figure 4:**
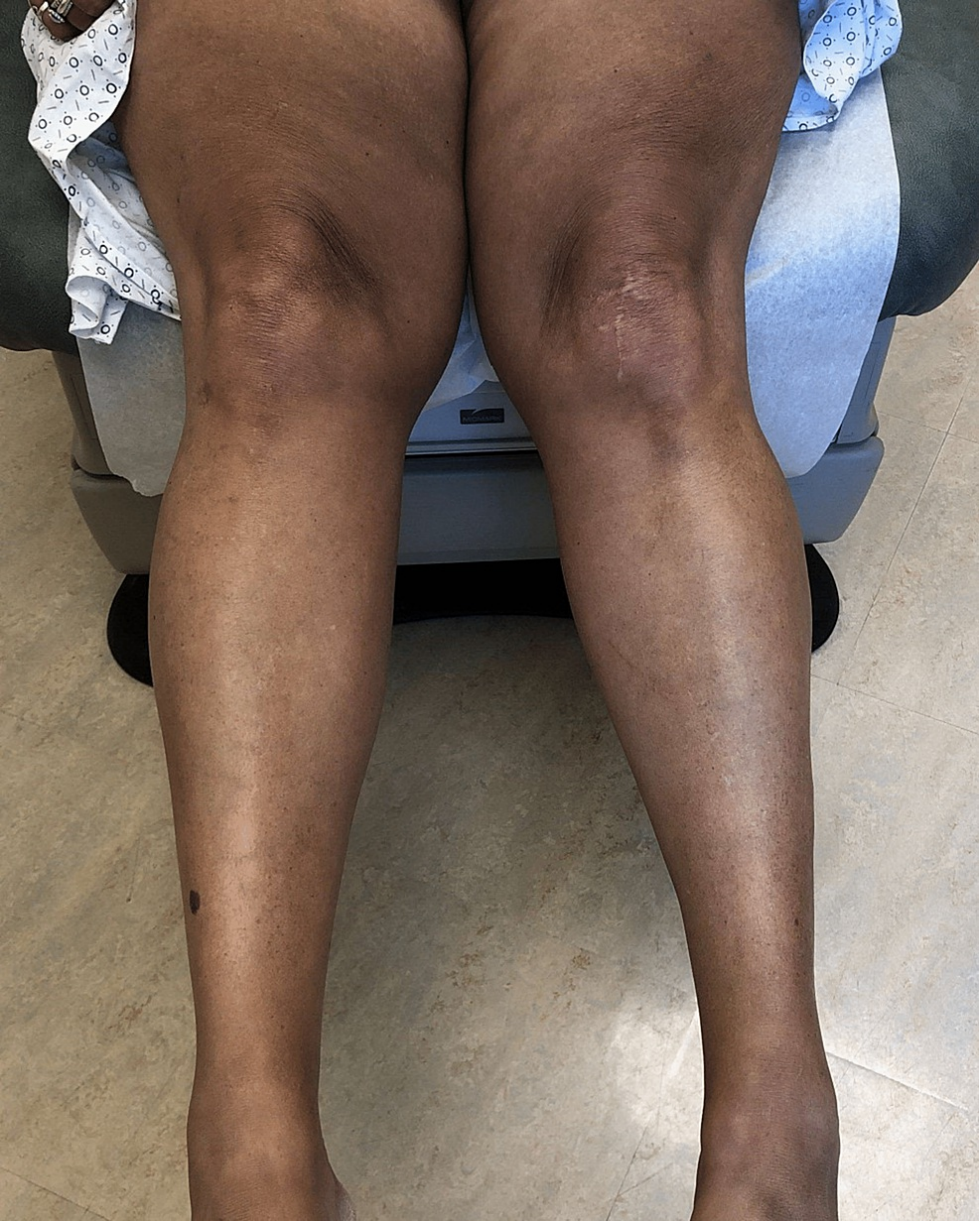
Hyperpigmented patches of bilateral thighs

**Figure 5:**
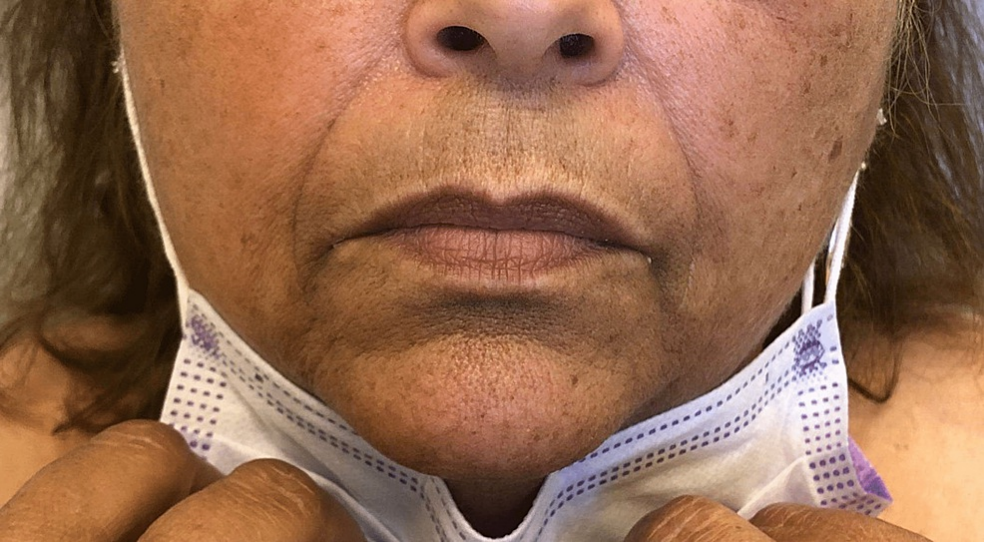
Hyperpigmented patch of the lower face

A 64-year-old female with a past medical history of invasive right breast adenocarcinoma with ductal and lobular features with metastasis to the axilla treated with anastrozole for 15 months and abemaciclib for 15 months, was admitted to the hospital for removal of an infected right breast implant and sepsis. She was started on piperacillin/tazobactam and vancomycin empirically on day one of her admission without any secondary red man syndrome from the vancomycin reported. The day after the removal of the infected implant, the patient developed acute-onset diffuse hyperpigmentation for which dermatology was consulted. The patient and her husband reported that she looked “tanner” than usual, but otherwise she was recovering well from her surgery and the hyperpigmentation was asymptomatic. She had not been treated with any medications containing silver or other heavy metals that she was aware of. The patient reported no similar episodes of hyperpigmentation in her past, no skin changes in general prior to admission, and no history overall of other skin conditions. She did note a history of fatigue and a recent weight gain of seven pounds. On exam, the patient presented with hyperpigmented patches in photodistributed areas including the face, neck, upper chest, arms, and legs (Figures 1-5).

Given the hyperpigmentation and reported history of fatigue, there was concern that the patient may have impending adrenal insufficiency from drug-induced Addison’s disease secondary to anastrozole. This prompted an endocrinology consult. The endocrinology team was reassured by the patient’s normotension, lack of hyperkalemia, hyponatremia, and no weight loss. The patient had subclinical hypothyroidism, but otherwise no personal or family history of autoimmune conditions. The patient's morning serum cortisol level was >14 and adrenocorticotropic hormone (ACTH) was within normal limits, ruling out adrenal insufficiency as the cause of her hyperpigmentation. 

The patient subsequently underwent a punch biopsy of the skin of her right thigh approximately two weeks after rash onset. The patient reported the hyperpigmentation had worsened since onset and may have slightly improved as she had been avoiding the sun. Differential diagnoses now included drug-induced hyperpigmentation, post-inflammatory hyperpigmentation, hemochromatosis, and phytophotodermatitis. The biopsy returned sparse superficial perivascular dermatitis with eosinophils, favoring a post-inflammatory hyperpigmentation reaction vs drug-induced hyperpigmentation.

Given this work-up and that hyperpigmentation is listed as a less common clinical trial adverse reaction in the abemaciclib product monograph, the hyperpigmentation was attributed to being most likely a result of this medication [1,2]. The vancomycin and piperacillin/tazobactam that she received inpatient was considered as a culprit, but we would have expected resolution of the hyperpigmentation since discharge and no further worsening of her condition. Further, these two antibiotics do not appear to be associated with hyperpigmentation in reviewing the literature. In addition, the iron stain was generally negative and serum ferritin was within normal levels, mitigating against a diagnosis of hemochromatosis. Phytophotodermatitis was also not consistent with histopathology. The patient elected to continue abemaciclib as her diffuse hyperpigmentation was not symptomatic. She continues to be followed by the dermatology department.

## Discussion

The CDK 4/6 inhibitors have made a significant impact on our approach to breast cancer treatment and are now commonly used, especially in advanced disease. They have shown remarkable efficacy, particularly in the treatment of hormone receptor-positive (HR+), human epidermal growth factor receptor negative (HER-) breast cancer [1]. They are important regulators of cell proliferation, migration, tumor angiogenesis, and overall inhibition of cell-cycle progression [3]. The most commonly reported side effect is bone marrow suppression, but cutaneous adverse events have also been reported with CDK 4/6 inhibitors and in anti-cancer treatments in general.

As CDK 4/6 inhibitor therapy becomes more commonly incorporated into breast cancer treatment regimens, there will likely continue to be an increase in reported cutaneous-related adverse effects from these targeted therapies. This is an important topic of study as these cutaneous manifestations can be diffuse and greatly influence a patient's quality of life and mental health [4]. A 2017 systemic review and meta-analysis of pigmentary changes associated with targeted anti-cancer treatments, in general, included 8,052 patients from 36 clinical trials [5]. The authors found that pigmentary changes were common over a patient's treatment course both in hypopigmentation and hyperpigmentation. Around 17.7% of patients experienced skin changes and 21.5% of patients had hair changes. The most commonly responsible agents were imatinib, cabozantinib, nivolumab, pazopanib, pembrolizumab, sorafenib, and sunitinib [5].

Specific and rare cases of dermatologic-associated CDK 4/6 inhibitor manifestations are vitiligo, toxic epidermal necrolysis, alopecia, acute bullous skin reaction, histiocytoid Sweet syndrome, and subacute cutaneous lupus [6,7]. These cutaneous adverse effects were not always evident on initiation of the medication; CDK4-6 inhibitor ribociclib-induced vitiligo was reported after five months of treatment [6]. Another recent case report of a 74-year-old patient experienced CDK4/6-induced subacute cutaneous lupus four months after a switch from palbociclib to abemaciclib for breast cancer treatment [7].

## Conclusions

Overall, cases of abemaciclib-associated dermatological adverse effects have not been widely reported and are minimally understood. Our goal with this case report is to continue to highlight the evolving literature on CDK4/6 inhibitor-associated cutaneous toxicities and contribute further information on possible abemaciclib-induced dermatological manifestations, specifically. In our case, the patient did not experience hyperpigmentation until after 15 months on abemaciclib. This does not appear to be unusual, as cutaneous side effects were not notable until months after initiating treatment with a CDK 4/6 inhibitor in multiple other case reports. 

In addition, we also completed a thorough review of the patient’s medications, none of which were associated with hyperpigmentation. Furthermore, we would have expected resolution of the patient’s skin condition upon discontinuation of certain medications (such as her vancomycin and piperacillin/tazobactam) at discharge. While abemaciclib-induced hyperpigmentation was a diagnosis of exclusion in this case, we feel this report is an important addition to the minimal literature on cutaneous reactions of CDK 4/6 inhibitors and helpful in understanding the possible effects of these newer agents that are increasingly being used in breast cancer treatment. It is possible that the stress of sepsis and hospitalization was the trigger for her onset of hyperpigmentation from the abemaciclib, although the reason for this timing is not something we are certain about. As noted earlier, these cutaneous toxicities can be widespread and have the potential to greatly affect patient quality of life, thus increasing the value of reporting these side effects and educating our patients to make informed decisions about their care.

## References

[1] Braal CL, Jongbloed EM, Wilting SM, Mathijssen RH, Koolen SL, Jager A (2021). Inhibiting CDK4/6 in breast cancer with palbociclib, ribociclib, and abemaciclib: similarities and differences. Drugs.

[2] (2022). Product monograph including patient medication information. https://pi.lilly.com/ca/verzenio-ca-pm.pdf.

[3] Kollmann K, Briand C, Bellutti F, Schicher N, Blunder S, Zojer M, Hoeller C (2019). The interplay of CDK4 and CDK6 in melanoma. Oncotarget.

[4] Silvestri M, Cristaudo A, Morrone A (2021). Emerging skin toxicities in patients with breast cancer treated with new cyclin-dependent kinase 4/6 inhibitors: a systematic review. Drug Saf.

[5] Dai J, Belum VR, Wu S, Sibaud V, Lacouture ME (2017). Pigmentary changes in patients treated with targeted anticancer agents: a systematic review and meta-analysis. J Am Acad Dermatol.

[6] Sharaf B, AlMasri R, Abdel-Razeq N, Salama O, Hamad I, Abunasser M, Abdel-Razeq H (2022). Vitiligo-like lesions in a patient with metastatic breast cancer treated with cyclin-dependent kinase (cdk) 4/6 inhibitor: a case report and literature review. Clin Cosmet Investig Dermatol.

[7] Kurtyka DJ, Mohebbi AD, Burke KT, Cardis MA (2021). Subacute cutaneous lupus erythematosus following abemaciclib therapy for metastatic breast cancer. JAAD Case Rep.

